# Hypoxia signaling in human diseases and therapeutic targets

**DOI:** 10.1038/s12276-019-0235-1

**Published:** 2019-06-20

**Authors:** Jae W. Lee, Junsuk Ko, Cynthia Ju, Holger K. Eltzschig

**Affiliations:** 1 Department of Biomedical Science, Graduate School, Kyung Hee University, Seoul, Republic of Korea; 2 Department of Biochemistry and Molecular Biology, MD Anderson UT Health Graduate School, The University of Texas Health Science Center at Houston, Houston, TX USA; 3 Department of Anesthesiology, The University of Texas Health Science Center at Houston, McGovern Medical School, Houston, TX USA

## Abstract

Since the discovery of hypoxia-inducible factor (HIF), numerous studies on the hypoxia signaling pathway have been performed. The role of HIF stabilization during hypoxia has been extended from the induction of a single gene erythropoietin to the upregulation of a couple of hundred downstream targets, which demonstrates the complexity and importance of the HIF signaling pathway. Accordingly, HIF and its downstream targets are emerging as novel therapeutic options to treat various organ injuries. In this review, we discuss the current understanding of HIF signaling in four different organ systems, including the heart, lung, liver, and kidney. We also discuss the divergent roles of HIF in acute and chronic disease conditions and their revealed functions. Finally, we introduce some of the efforts that are being performed to translate our current knowledge in hypoxia signaling to clinical medicine.

## Low oxygen: Protein signalling in human disease

A protein family that plays diverse roles in acute and chronic hypoxia (low oxygen) may prove valuable in developing novel therapies for different diseases. Hypoxia, the short- or long-term depletion of oxygen in cells, tissues and organs, is involved in many disease conditions. Holger Eltzschig at the McGovern Medical School in Houston, USA, and co-workers reviewed research into the roles of a key hypoxia regulator, the hypoxia-inducible factor (HIF) protein group, in diseases affecting the heart, lung, liver and kidneys. HIF protein activity is often beneficial in acute conditions such as myocardial ischemia, sending signals to protect cells and increasing tissue tolerance to hypoxia. However, during chronic hypoxia, HIF signaling may contribute to the progression of disease, for example in pulmonary fibrosis. HIF stabilizers and HIF-induced targets as drugs could prove valuable in future treatments.

## Introduction

**Fig. 1:**
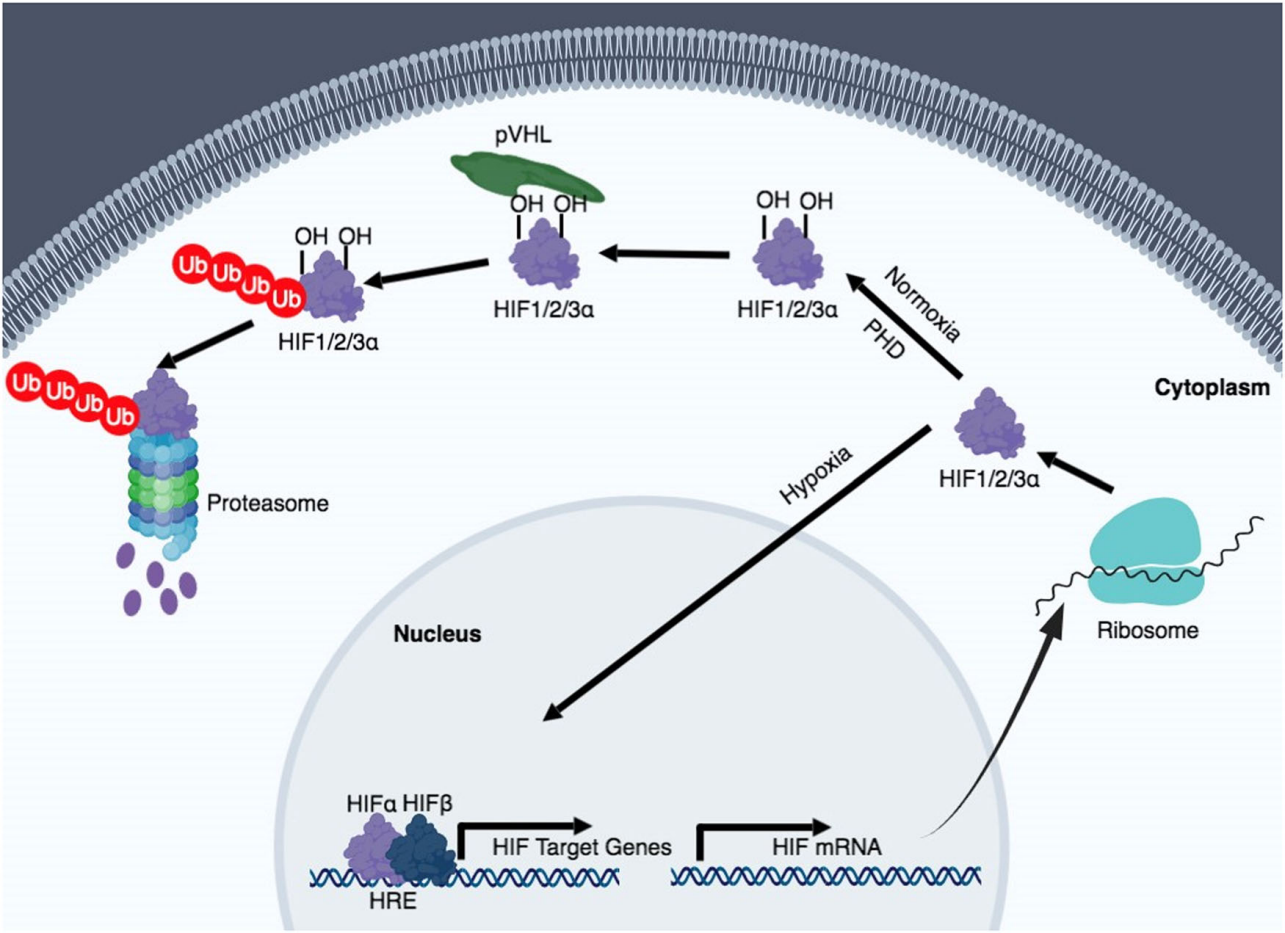
Hypoxia-inducible factor (HIF) regulation during normoxia and hypoxia. In oxygenated conditions, HIF is hydroxylated on proline residues by prolyl-4-hydroxylases (PHDs) and polyubiquitinated by the von Hippel–Lindau protein (pVHL). This leads to degradation of HIF by the 26S proteasome system. In hypoxic conditions, HIF is stabilized and translocated into the nucleus, where it binds to its dimerization partner HIF1B and enhances the transcription of HIF target genes

Hypoxia refers to a condition in which oxygen is limited. It is mainly related to pathological situations, but it can also be a part of normal physiology. In humans, oxygen is exchanged in the alveoli of the lungs. Over 95% of oxygen that is delivered into the capillary vessels through the alveolar–capillary exchange system binds to hemoglobin. The heart pumps oxygenated blood to the periphery, which is crucial for organs and cells to function and perform oxidative phosphorylation. Hypoxia can result from any failure that might happen during this process, which encompasses failure of the respiratory system, insufficient blood flow to an end organ, dysfunctional or low levels of hemoglobin, or chemically induced hypoxia. Hypoxia activates the hypoxia signaling pathway, which is predominantly governed by hypoxia-inducible factor (HIF) stabilization (Fig. 1). In normoxic conditions, the proline residues of HIF-α subunits are hydroxylated by oxygen-dependent prolyl-4-hydroxylases (PHDs). Von Hippel–Lindau protein (pVHL), an E3 ubiquitin ligase, binds to the hydroxylated HIF-α and acts as a substrate recognition component of the E3 ubiquitin ligase complex, which leads to the proteosomal degradation of HIF protein. The asparagine residues of HIF-α subunits are also hydroxylated by factors inhibiting HIFs (FIHs), which inhibits the binding of HIF with co-activators p300/CREB-binding protein. Under hypoxia, the activity of PHDs and FIHs are suppressed, and HIF-α subunits translocate into the nucleus to bind with HIF-1β (HIF1B). The heterodimeric HIF-α: HIF-1β transcription factor complex then locate to the hypoxia-responsive elements (HREs) of its target genes, resulting in their transcriptional upregulation. There are other HIF-independent signaling pathways that are activated under hypoxia, such as the nuclear factor-κB (NF-κB) pathway. Early studies reported that IκBα was phosphorylated during hypoxia and this results in the degradation of IκBα and the activation of NF-κB^1^. Another study showed that IκB kinase activity is increased through calcium/calmodulin-dependent kinase 2 during hypoxia and transforming growth factor-β (TGF-β)-activated kinase 1 is required^2^. All of these studies support the notion that hypoxia and inflammation have an interdependent relationship^3,4^. In fact, many studies demonstrate that although hypoxia can cause tissue inflammation, stabilization of HIF can dampen tissue inflammation and promote its repair^5–8^.

**Fig. 2:**
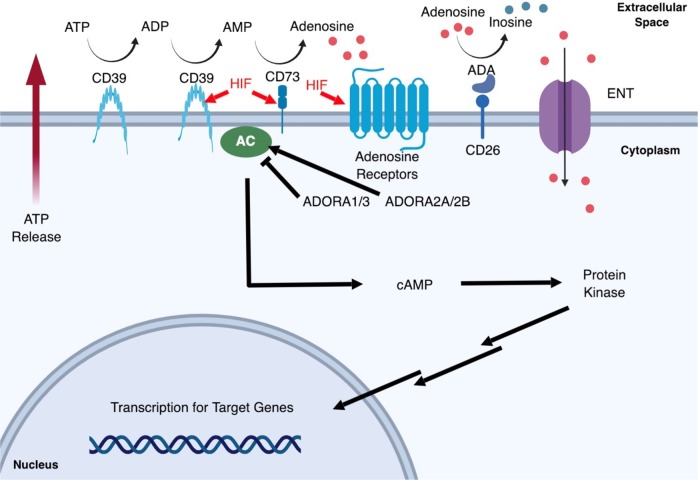
Adenosine signaling pathway. During the hypoxic insult, cells release adenosine triphosphate/adenosine diphosphate (ATP/ADP) that accumulate in the extracellular space. Hypoxia triggers the SP1-dependent induction of CD39 and HIF-dependent induction of CD73, which converts ATP to ADP, AMP, and eventually adenosine. HIF also upregulates adenosine receptor levels and together with the increased extracellular adenosine, the downstream purinergic signaling pathway is activated. Extracellular adenosine could re-enter into the cell by equilibrative nucleoside transporters (ENTs) or could be deaminated by CD26-conjugated adenosine deaminases (ADAs), all of which function to terminate adenosine signaling. Stimulation of adenosine receptors either result in the inhibition of adenylyl cyclase (AC) by ADORA1/3 or activation by ADORA2A/2B

What are the results of HIF stabilization during hypoxic conditions? HIF elicits a wide range of adaptive responses, which mainly focus on the upregulation of transcriptional cascades that are important for tissue protection and adaptation. HIF-1α (HIF1A) is known to be associated with the upregulation of glycolytic genes such as phosphoglycerate kinase (*PGK*) and lactate dehydrogenase A (*LDHA*), both of which function to metabolically adapt the tissue to oxygen deprivation and anaerobic ATP synthesis. HIF-2α (HIF2A) induces erythropoietin (*EPO*) and vascular endothelial growth factor (*VEGF*), which are important to improve oxygen supply to the hypoxic region^9^. A genome-wide association study to profile the targets of HIF1A and HIF2A, respectively, showed that both bind to an identical core-binding motif also known as the HRE (5′-RCGTG-3′) and share multiple binding sites throughout the genome. HIF-dependent upregulation of certain genes are common, but HIF-dependent suppression of genes are unusual. In spite of the fact that HIF1A and HIF2A bind to similar sites with similar affinity, HIF1A contributes more to the acute hypoxia-driven transcriptional responses^10^. A remarkable example of HIF-driven protective response is demonstrated through the link between HIF and adenosine (Fig. 2)^5,11^. Upon hypoxic cellular and tissue injury, extracellular ATP/ADP is accumulated. Stabilized HIF1A binds to the promotor region of *CD73* and increases ecto-5′-nucleotidase (CD73) enzyme levels, which in turn increases adenosine levels^12^. Unlike intracellular adenosine, extracellular adenosine can directly act as a signaling molecule through adenosine receptors. Other key regulators of adenosine signaling are also direct targets of HIF: adenosine receptor 2B (ADORA2B) by HIF1A and adenosine receptor 2A (ADORA2A) by HIF2A^13^. Indeed, increasing extracellular adenosine levels by the inhibition of equilibrative nucleoside transporters result in the dampening of inflammation^14^. Together, the adenosine signaling pathway serves as a protective mechanism and provides ischemic tolerance in tissues exposed to acute hypoxia.

In this review, we will discuss the current understandings of hypoxia signaling in human diseases by the organ systems, including the heart, lung, liver, and kidney. We will also discuss the potential therapeutic targets that may serve as novel treatment options.

## Heart disease

### Ischemic heart disease

Ischemic heart disease is caused by the narrowing of a major coronary artery or the rupture of coronary plaque, resulting in either less blood flow or decreased oxygen supplement to the cardiac muscles. This leads to decreased ATP production, dysfunction of pumps, and cell death within the ischemic region. The current treatment strategy is emergent reperfusion of the ischemic myocardium, either by percutaneous coronary intervention or intravenous fibrinolytic therapy. Restoration of blood flow causes the generation of reactive oxygen species and activation of inflammatory cascades, which is also known as ischemia reperfusion injury (IRI)^15^. Numerous studies have shown cardio-protective mechanisms by the induction of HIFs during ischemic conditions. HIF1A is reported to increase in peri-infarct areas of human ischemic heart tissues^16^. Similarly, HIF2A is increased in the mouse heart after coronary artery occlusion, 8% oxygen (normobaric hypoxia), or 0.1% carbon monoxide exposure^17^. These studies demonstrate that HIF is increased in the heart during hypoxic conditions. The role of increased HIF has been reported to be critical in conveying cardio-protection. HIF1A increase inducible NO synthase levels, which dampens ischemic injury^18^. An in vivo rabbit model of IRI shows that by the activation of HIF1A, its downstream target heme oxygenase 1 (HO-1) is induced and this attenuates proinflammatory cytokine production^19^. Notably, HIF is a central component of ischemic preconditioning (IPC) in the heart. IPC is an experimental technique in which short, repetitive episodes of ischemia and reperfusion before the subsequent long exposure to ischemia pre-adapts the myocardium to protective mechanisms. HIF1A knockdown by small interfering RNA abolishes the protective effects of IPC, whereas PHD2 knockdown or HIF activator dimethyloxalylglycine (DMOG) treatment mimic the effects of IPC^20^. The effect of HIF1A is explained by the induction of *CD73*, increased adenosine, and upregulated ADORA2B levels^21^. HIF2A stabilization during preconditioning is shown to have a role in increasing cardiomyocyte resistance to hypoxia. Β_1_-subunit gene (*KCNMB1*) is repressed by HIF2A stabilization, which leads to increased cell viability during the simulated ischemia after preconditioning^22^. HIF is also implicated to have a role in ischemic postconditioning (IPostC). Compared with IPC, IPostC is carried out by applying short ischemia/reperfusion cycles right after the ischemic event. It is clinically more relevant, because patients come in the hospital during the ischemic attack—not before the event happens. HIF1A is increased after IPostC, which correlates to the reduction in infarct size. When DMOG is given along with the IPostC protocol, infarct size and caspase-3 activity are both dampened^23^. Most recent studies emphasized the role of HIF2A as the key transcription factor in conveying cardio-protection during IRI. Myocyte-specific deletion of HIF2A and not HIF1A shows that HIF2A is important in increasing ischemic tolerance by upregulating its target gene amphiregulin and activating downstream AKT signaling pathway^24^. Taken together, HIF stabilization during acute myocardial ischemia and reperfusion injury provides tissue tolerance to ischemic injury.

### Congestive heart failure

Congestive heart failure (CHF) is the failure of the heart to maintain its contractility, leading to ineffective blood flow to peripheral organs and signs of dyspnea and fatigue. Common causes for this condition are coronary artery disease, hypertension, and cardiomyopathy. Pressure overload and increased heart rate puts more burden to the myocardium, which worsens the mismatch between oxygen demand and supply. This leads to chronically activated hypoxic responses in the heart^25^. First-line medication include angiotensin-converting enzyme inhibitors (ACEIs) or angiotensin receptor blockers (ARBs) and β-adrenergic blocking agents, which improve symptoms and reduce mortality. In contrast to the acute protective roles of HIF, chronic HIF stabilization has shown to have detrimental effects. HIF1A transgenic mice develop spontaneous cardiac hypertrophy with age. Transverse aortic constriction (TAC) surgery at a younger age shows a significant increase in left ventricle end-systolic diameter^26^. This is explained by the induction of several glycolytic enzymes and downregulation of the sarcoplasmic endoplasmic reticulum calcium ATPase pump, which results in cardiac dysfunction^27^. In a rat model of aorta-caval shunt to induce volume-overload heart failure, HIF1A and VEGF expression were increased. Carvedilol, a β-blocker to treat heart failure, reversed the abnormal regulation of HIF1A and VEGF^28^. Another independent study shows that HIF1A is necessary for the myocardium to adapt to pressure overload and increase angiogenesis. Increased HIF1A is beneficial; however, the accumulation of p53 from prolonged pressure overload inhibits the HIF1A activity and impairs cardiac angiogenesis and systolic function, thus leading to heart failure^29^. A recent study demonstrated that during hypoxia, HIF1A upregulates the expression of splice factor 3b subunit 1. As a consequence, the alternative splicing pattern of fructose-metabolizing enzyme ketohexokinase (KHK) changes and *Khk*-C mRNA, but not *Khk*-A mRNA, levels increase. Knockout of both *Khk*-A and *Khk*-C prevents pathologic cardiac hypertrophy and prevents the promotion of anabolic metabolism^30^. Relative to HIF1A, there are not many studies related to HIF2A. Adipocyte-specific HIF2A deletion was reported to revert the lethal cardiomegaly that was observed in homozygous adipocyte-specific VHL knockout mice, indicating that adipocyte HIF2A is required for the development of adipocyte-induced cardiac hypertrophy^31^.

### Valvular heart disease

Valvular heart disease is a disease that occurs in one of the four valves of the heart, which is usually presented as narrowing (stenosis) or insufficiency to shut properly (regurgitation). It is mainly caused by degeneration of tissue as a result of aging, but there are other factors such as rheumatic heart disease or congenital defects. Aortic stenosis is the most common type, followed by mitral regurgitation. HIF has been implicated in the development of the heart valve. VEGF is a regulator of endothelial cell proliferation and its spatiotemporal expression is important for the endothelial cells to undergo endothelial-mesenchymal transdifferentiation (EndMT). Pathological levels of hypoxia or hyperglycemia leads to altered levels of VEGF, thus contributing to congenital cardiac defects^32^. Previous studies have shown a correlation between valvular disease and hypoxia signaling. In some regions of the aortic valve, the diffusion of oxygen is insufficient. Hypoxia in mitral valve interstitial cells results in the stabilization of HIF1A, increase of matrix remodeling enzyme gene expression, such as matrix metallopeptidase-2 and -9 (*MMP2* and *MMP9*), and the reduction of collagen production and sulfated glycosaminoglycans (sGAG) levels^33^. NF-κB and HIF2A expression is increased in the valve leaflets of patients with aortic stenosis. Expression of VEGF, neoangiogenesis, and collagen X are observed in areas where NF-κB and HIF2A are co-localized, which demonstrates that these signaling pathways could contribute to the pathophysiology of aortic stenosis^34^.

## Pulmonary disease

### Acute lung injury

Acute lung injury (ALI) is an inflammatory lung disease, which is characterized by pulmonary edema and significant hypoxemia. The pulmonary edema attributes to the increased permeability in endothelial cells, allowing infiltration of protein-rich fluid and immune cells into parenchyma. The accumulated fluid in the alveoli significantly lowers the efficacy of air exchange between the alveoli and vasculature, leading to hypoxemia and regional alveolar hypoxia. The major risk factors for ALI are sepsis, severe traumatic injury, and cigarette smoking. Treatment options are lung-protective mechanical ventilation and supportive management. Although there is no evidence that hypoxia is the direct cause of ALI, some studies suggest that hypoxia may contribute to the pathogenesis of ALI. Acute exposure to hypoxia alone can cause ALI-like phenotypes, such as increased vascular leakage and infiltration of immune cells in the lungs of the rodents, suggesting that regional alveolar hypoxia established by lung injury may perpetuate the injury phenotypes^35,36^. On the other hand, the stabilization of HIF during hypoxia has been shown to be protective during ALI^37^. DMOG treatment attenuates pulmonary edema and lung inflammation during the ventilation-induced lung injury, and inhibition of HIF1A via echinomycin aggravates lung injury. Whole-body and alveolar epithelial cell-specific HIF1A knockdown mice exhibited decreased survival time, increased pulmonary edema, and attenuated gas exchange during ALI, revealing that HIF1A is crucial in dampening lung inflammation in vivo^38^. Moreover, HIF1A is known to transcriptionally target *Adora2b* to exert its protective effect during ALI^39,40^. HIF1A has also been implicated in promoting the repair of the alveolar epithelium after ALI. HIF is activated in alveolar type 2 (ATII) cells after ALI, which then promotes ATII cells spreading by increased SDF1 and CXCR4^41^. Although conditional deletion of HIF1A in myeloid cells does not show protection in ventilator-induced ALI^38^, HIF1A stabilization in myeloid cells shows a pathological effect in the lipopolysaccharide (LPS)-induced ALI^42^. This inconsistency is presumably due to the differences in the ALI models. In contrast to HIF1A, HIF2A is relatively understudied for ALI; only one study clearly shows the direct role of HIF2A in ALI^43^. This study reveals that vascular endothelial protein tyrosine phosphatase (VE-PTP), a key regulator of endothelial barrier integrity, is a transcriptional target of HIF2A, and that HIF2A deletion in endothelial cells of mice exacerbates LPS-induced ALI, presumably due to the lack of VE-PTP regulation.

### Pulmonary hypertension

Pulmonary hypertension (PH) is a condition characterized by high blood pressure in the arteries of the lung. The World Health Organization has classified PH into five subgroups, of which group 1 is idiopathic or heritable and group 3 is PH due to lung disease or chronic hypoxia. The cause is unclear and patients present insidious symptoms such as increasing fatigue or dyspnea. In PH, pulmonary artery endothelial cells (PAECs) are altered and release factors that stimulate smooth muscle cell proliferation, which leads to neointimal formation and progressive thickening of arteries. Dysregulated, proliferating PAECs also exhibit increased synthesis of vasoconstrictors and decreased amounts of nitric oxide (NO) and prostacyclin^44^. HIF has been reported to contribute to the pathogenesis of PH. Both *Hif1α*^+/−^ and *Hif2α*^+/−^ mice have been shown to be protected from developing chronic hypoxia induced PH^45,46^. The increase in endothelin-1 and catecholamine levels is dampened in *Hif2α*^+/−^ mice, indicating that they are downstream targets of HIF2A during chronic hypoxia^46^. Ball et al.^47^ showed that smooth muscle-specific knockdown of HIF1A attenuated pulmonary vascular remodeling and PH but did not affect the hypertrophic changes that occur in the right ventricle. Recent studies show that endothelial cell-specific knockdown of HIF2A prevents mice from developing hypoxia-induced PH. Increased HIF2A levels enhance EndMT and upregulate zinc finger protein SNAI1/2 expression, resulting in vascular remodeling^48^. Collectively, these studies suggest that HIF stabilization during PH contributes to the pathogenesis and inhibiting HIF could be a therapeutic target.

### Pulmonary fibrosis

Pulmonary fibrosis (PF) is a chronic interstitial lung disease characterized by aberrant wound-healing and excessive scarring in the parenchyma. Among the sub-types of PF, idiopathic PF (IPF) is the most common and severe type of lung fibrosis with unknown cause. The current understanding of the mechanism of IPF is that repeated initial injuries in the epithelial cells cause abnormal wound-healing in ATII epithelial cells by recruiting immune cells and ultimately activating fibroblasts to produce extracellular matrix proteins, such as fibronectin and collagen^49^. These pathological matrix proteins remodel the lung structures, which impair the air exchange between the parenchyma and vasculature, leading to regional hypoxia in fibrotic areas and ultimately systemic hypoxemia^50^. Currently, two drugs have been approved by the FDA for use in PF patients. Pirfenidone, which has anti-fibrotic and anti-inflammatory properties, is known to improve progression-free survival. Nintedanib inhibits receptors related to the pathogenesis of PF, such as the platelet-derived growth factor (PDGF) receptor, fibroblast growth factor (FGF) receptor, and VEGF receptor, and decelerates the decrease in forced vital capacity. HIF has been implicated in the pathogenesis of PF. HIF1A is stabilized in many cell types in IPF lungs, such as ATII cells and the cells in the fibrotic area^51^. TGF-β1, which is the most potent inducer of fibrogenesis, inhibits PHD2 expression and induces HIF1A stabilization in fibroblasts^52^, suggesting that the HIF signaling pathway can also be activated without prominent hypoxic conditions. Using two independent Cre systems, a recent study further demonstrated that HIF1A deletion in lung fibroblasts attenuated the bleomycin-induced PF^53^. For detailed mechanisms in fibroblasts, it has been suggested that HIF1A targets pyruvate dehydrogenase kinase to switch the glucose metabolism of the cells to glycolysis, leading to myofibroblast differentiation. In addition to fibroblasts, HIF1A also has a pathological role in macrophages; HIF1A induced by hypoxia promotes the fibrotic phenotype of alternatively activated macrophages through increased ADORA2B expression, cell differentiation, and production of profibrotic mediators, such as interleukin-6^54,55^. Tanjore and colleagues^51^ recently reported that HIF1A and HIF2A double knockout in ATII cells does not show protection against lung fibrosis induced by intratracheal injection of bleomycin in mice. This result suggests that HIF signaling may not have important roles in ATII during the initial developmental steps of PF, but may act as an amplifier of the disease in fibroblasts.

### Chronic obstructive pulmonary disease

Chronic obstructive pulmonary disease (COPD) is a progressive obstructive lung disease that presents as wheezing and productive cough. Emphysema and airflow limitation caused by the remodeling of lung structure result in ventilation/perfusion mismatch, leading to hypoxic conditions. The major risk factors associated with COPD are chronic cigarette smoke exposure and air pollution. COPD is managed by bronchodilators such as β_2_ agonists, anticholinergics, and corticosteroids during acute exacerbations. Although not all COPD patients have a history of smoking, it is evident that COPD patients who smoke have significantly worse lung injuries. There are a number of studies demonstrating that primary and secondary exposures to cigarette smoke cause COPD-like lung damages in mouse models, such as emphysema, vascular remodeling, and excessive epithelial apoptosis^56^. VEGF signaling is significantly impaired in rodents exposed to cigarette smoke, leading to excessive epithelial and endothelial cell apoptosis^57^. HIF1A has a detrimental role for mucus hypersecretion caused by cigarette smoke exposure, which is one of the complications of COPD. Following the treatment of cigarette smoke extract, HIF1A transcriptionally targets mucin 5AC in airway epithelial cells, causing mucin overproduction^58^. HIF1A has also been implicated to increase deoxycytidine kinase (*DCK*), an enzyme that phosphorylates deoxyadenosine and increases the accumulation of deoxyATP. COPD patients have significantly increased levels of DCK, which in turn contributes to elevated deoxyATP levels, increased apoptosis, and disease amplification^59^. In contrast to the above mentioned studies, Mizuno et al.^60^ showed that patients with severe COPD, classified by the Global Initiative for Chronic Obstructive Lung Disease, have lower levels of HIF1A and VEGF, and higher levels of miR-34a and miR-199a. Oxidative stress induces p53 and miR-34a, leading to AKT inactivation. In turn, inactivated AKT leads to the upregulation of miR-199a, which inhibits HIF1A and VEGF levels, resulting in impaired lung maintenance^60^. Together, these studies demonstrate the complex role of HIF in the pathogenesis of COPD.

## Liver disease

### Acute liver failure

Acute liver failure (ALF) is the rapid loss of liver function characterized by increased serum alanine and aspartate aminotransferase levels, prothrombin time, and serum bilirubin. Common causes for this condition are acetaminophen (APAP) overdose, excessive alcohol consumption, acute viral hepatitis, and IRI. Treatment is focused on reversing APAP overdose by *N*-acetylcysteine or providing supportive management until the liver function returns back to normal. HIF induction has been reported to be protective during ALF. HIF1A is upregulated during thioacetamide (TAA)-induced ALF in mice along with its downstream target CD73. DMOG treatment before TAA treatment dampens liver injury^61^. HIF1A is also stabilized in the liver with severe APAP injury, which stimulates the cleavage of hemojuvelin, a cofactor of bone morphogenic protein, thereby downregulating the expression of hepcidin^62^. T-cell-specific deletion of HIF1A worsens APAP-induced acute liver injury by increasing hepatic accumulation of innate-like γδ T-cells and neutrophils^63^. HIF has also been shown to be beneficial during hepatic IRI injury. IRI injury is caused by the re-oxygenation of a previously ischemic hepatic tissue, which occurs frequently during hemorrhagic shock, surgery, and transplantations. In response to IRI, sinusoidal microvasculature constricts and entraps leukocytes, leading to further impaired blood flow and tissue hypoxia^64^. Pharmacological induction of HIF1A ameliorates the IR liver injuries in rodents by upregulating HIF1A-dependent antioxidant genes and reducing mitochondrial damage^65–67^. In addition to the pharmacological interventions, a transgenic mouse model lacking PHD1 (therefore activating HIF) displays remarkable tolerance to acute hypoxia and protection against IR liver injury^68^. Another independent study unveiled the protective function of HIF by demonstrating that PHD deletion promoted liver regeneration following a hepatectomy of 80%^69^. Similar to the heart, IPostC, after an ischemic insult by repeated occlusion and reopening of the portal vein, is protective in that it increases NO and HIF1A levels, and decreases tumor necrosis factor-α and intercellular adhesion molecule 1 levels^70^. HIF is also known to target several genes involved in the extracellular adenosine signaling^71^. Upon IR liver injury, stabilized HIF directly targets *CD73* to upregulate extracellular adenosine and enhance adenosine signaling through ADORA1 and ADORA2A, thereby exerting the protective function against IR damage^72–74^. The ADORA2A markedly inhibits the production of interferon-γ by natural killer T cells, which dampens hepatic IRI injury.

### Liver fibrosis and cirrhosis

Liver fibrosis is the scarring of the liver tissue caused by chronic liver injuries following viral infection, alcohol intake, or environmental toxins. This is a natural wound-healing process. However, repeated insults can trigger an uncontrolled abnormal response to injury that leads to the activation of hepatic stellate cells and overexpression of matrix proteins^75^. Advanced liver fibrosis can further progress to cirrhosis where the functional units of the liver are irreversibly replaced by the extracellular matrix proteins. Current management options include symptomatic alleviation by removing excess fluid from the body or administering antibiotics and lactulose, but the only curative method is liver transplantation. The pathological role of HIF1A has been well documented in the pathogenesis of liver fibrosis. HIF1A-deficient mice display reduced liver fibrosis upon injury caused by bile duct ligation (BDL), which is an established model of liver fibrosis^76^. The protective role of HIF1A deletion in this study is associated with the reduction of profibrotic mediator levels, such as PDGFs, FGFs, and connective tissue growth factors, which are well-known targets of HIF1A^77^. Another independent study demonstrated that conditional deletion of HIF1A in hepatocytes attenuates liver fibrosis induced by a high *trans*-fat diet in mice, which is another model of liver fibrosis^78^. Collectively, these studies suggest that HIF stabilization by regional hypoxia or oxygen-independent stimuli, such as cytokines or ROS, transcriptionally activates the gene expression of profibrotic mediators leading to the progression of liver fibrosis^77^. In addition to hepatocytes as the regulator of HIF-mediated liver fibrosis, myeloid cells may have pivotal roles. The deficiency of HIF1A and HIF1B in macrophages significantly suppress PDGF-β mRNAs and proteins, thus reducing α-smooth muscle actin and type 1 collagen levels in BDL models^79^. In hepatic stellate cells, which are a major factor for matrix protein synthesis, essential genes for angiogenesis, collagen synthesis, and chemotaxis are upregulated during hypoxia via HIF1A stabilization^80^. The broad effect of HIF during liver fibrosis suggests that this pathological pathway can be regulated at multiple levels. HIF1A itself can be targeted for therapeutic purposes. A recent study demonstrates that inhibition of hypoxia signaling via overexpression of pVHL, a negative regulator of HIF, can significantly attenuate liver fibrosis induced by both the BDL and CCl_4_ administration. Furthermore, the protective role of VHL is reduced by the overexpression of HIF1A and HIF2A in vivo, indicating that the VHL protection is mediated through HIF inhibition^81^.

## Kidney disease

### Acute kidney injury

Acute kidney injury (AKI) is an abrupt loss of kidney function that is diagnosed by decreased urine output or increased urea and creatinine. AKI is commonly classified into three categories based on the etiologies: prerenal, intrinsic, and postrenal. The most common form of AKI in hospital in-patients in developed countries is prerenal, which is mainly caused by decreased effective blood flow to the kidneys. Depending on its etiology, AKI is treated with intravenous fluids, diuretics, and hemodynamic support. HIF activation has been reported in renal ischemia models and cisplatin- or gentamicin-induced renal injury models. Cisplatin itself can reduce renal blood flow and induce subsequent hypoxia in the outer medulla region, contributing to the damage of tubular cells^82,83^. HIF1A is mainly induced in the papillary tubular cells, whereas HIF2A is induced more frequently in the glomeruli and peritubular interstitium. HIF1A shows co-localization with HO-1 and glucose transporter-1, and HIF2A with EPO, suggesting a selective induction and distinct roles of each HIF subtype^84^. HIF activation has been shown to have an important role in providing renal protection during AKI. In the experimental mouse model of gentamicin-induced AKI, HIF1A expression significantly increases and correlates with reduced caspase-3 activity, histologic injury, and macrophage infiltration^83^. Similar to the protective effects of IPC in the heart, repetitive hypoxic preconditioning increases renal HIF1A mRNA and protein levels, and decreases mitochondrial Bax translocation, cytochrome *c* release, and tubular apoptosis^85^. Another independent study showed that hypoxic preconditioning reduces cisplatin-induced apoptosis in the tubular cells^82^. Similar to the reno-protective role of HIF1A, HIF2A is also shown to be important in providing these protective effects. Both *Hif1α*^+/−^ mice and *Hif2α*^+/−^ mice show more severe histologic damage after renal IRI and treatment with DMOG attenuates IRI-induced renal injury^86^. Mice with endothelial-specific deletion of HIF2A develop increased renal injury markers and inflammatory cell infiltration during ischemic injury compared with wild-type mice. Furthermore, treatment of HIF2A endothelial-specific knockout mice with HIF prolyl-hydroxylase inhibitors show no protective effects^87^. Together, these studies support a reno-protective role of HIF stabilization during AKI.

### Chronic kidney disease

Chronic kidney disease (CKD) is characterized by the progressive loss of kidney function, which initially manifests with no specific symptoms but later progresses with various signs of kidney failure including increased blood pressure, accumulation of urea, anemia, hyperkalemia, fluid volume overload, hyperphosphatemia, hypocalcemia, and metabolic acidosis. The severity of impairment in CKD correlates with histologic features such as inflammation, extracellular matrix accumulation, atrophy of tubular structures, and peritubular capillaries^88^. Common causes of CKD includes diabetes mellitus, hypertension, and glomerulonephritis. The goal of therapy is to relieve symptoms and decelerate the progression of disease. ACEIs or ARBs are used in this context; however, renal replacement therapy such as dialysis or kidney transplant are eventually required. The contribution of hypoxia to CKD has been hypothesized and supported by several studies. Primary glomerular injury results in altered dynamics of the postglomerular peritubular regions and consequent hypoxia in the related region. This prompts tubular injury, EndMT, inflammation and recruitment of circulating cells, myofibroblast differentiation, and interstitial fibrosis^88^. HIF1A is increased in renal biopsies of diabetic nephropathy patients and enhances EndMT through the upregulation of lysyl oxidase genes^89^. HIF1A activates the transcription of *COL1A2*, which requires SMAD3 and is enhanced by TGF-β^90^. Deletion of pVHL in intrinsic glomerular cells of mice initiates necrotizing crescentic glomerulonephritis by the increase of the HIF target gene *Cxcr4*^91^. Epithelial HIF1A deletion inhibits the development of tubulointerstitial fibrosis and silencing of HIF1A attenuates angiotensin II-induced renal injury^92^. Recent studies show a previously unrecognized role of HIF2A stabilization in the kidney epithelium. EPO is known to be synthesized in peritubular interstitial fibroblast-like cells by the induction of HIF2A. However, HIF stabilization in the proximal tubular epithelial cells suppress EPO production by inhibiting the conversion of non-EPO-producing peritubular interstitial cells to renal EPO-producing cells^93^.

## Potential targets for therapeutic intervention

In the above paragraphs, we have discussed different organ systems, diseases, and how hypoxia signaling is involved in the process. HIF seems to have contrary effects: one being protective during acute settings and the other being injurious and contributing to the pathophysiology of disease in chronic settings (Table 1). During acute hypoxic conditions, HIF is rapidly stabilized and its downstream targets are promptly increased. These target genes are directly related to metabolism, proliferation, survival, and angiogenesis, which provide ischemic tolerance. Conversely, in chronic conditions such as CHF, lung fibrosis, liver fibrosis, and chronic kidney injury, HIF is engaged in the pathogenesis of the disease. The adverse effects of chronic HIF stabilization in diseases are summarized in Table 1. As chronic diseases take time to develop and are the result of multiple etiologies, the precise spatiotemporal involvement of HIF and its role is difficult to delineate. As such, the function of HIF is controversial in different studies. For example, the role of HIF1A in a TAC model to mimic heart failure produced disparate results. One study reported that cardiac-specific overexpression of HIF1A aggravates heart failure^26^, the other study showed that HIF1A and its contribution to angiogenesis is an important adaptive mechanism during cardiac hypertrophy^29^. Moreover, endothelial HIF1A knockdown in the heart increased TGF-β signaling, thus leading to pathological remodeling^94^. These results raise the possibility that each cell type within the organ may have different properties of HIF activation and their roles. Indeed, more studies focusing on the role of HIF in different cell types and different organs are being conducted^95^.

**Table 1:** HIF stabilization and its downstream targets in different organ systems

Organ		Experimental model	Subtype	Downstream target	Adverse effect
Heart	Acute	Ischemic preconditioning	↑HIF1A	↑CD73^20^	
			↑HIF1A	↑ADORA2B^21^	
			↑HIF2A	↓KCNMB1^22^	
		Ischemia/reperfusion	↑HIF1A	↑iNOS^18^	
			↑HIF1A	↑HO-1^19^	
			↑HIF2A	↑AREG^24^	
	Chronic	Aorta-caval shunt	↑HIF1A	↑VEGF^28^	Adverse cardiac remodeling
		Transverse aortic constriction	↑HIF1A	↑SF3B1, ↑KHK-C^30^	Promotion of anabolic metabolism
Lung	Acute	Ventilator-induced lung injury	↑HIF1A	↑ADORA2B^39^	
		CLP-induced lung injury	↑HIF2A	↑VE-PTP^43^	
	Chronic	Chronic hypoxia	↑HIF2A	↑ET1^46^	Pulmonary hypertension
			↑HIF2A	↑SNAI1/2^48^	Vascular remodeling
		Bleomycin-induced pulmonary fibrosis	↑HIF1A	↑PDK1^53^	Myofibroblast differentiation
		Cigarette smoke-induced pulmonary injury	↑HIF1A	↑MUC5AC^58^	Mucous hypersecretion
Liver	Acute	TAA-induced liver injury	↑HIF1A	↑CD73^61^	
		APAP-induced liver injury	↑HIF1A	↑Cleavage of hemojuvelin, ↓Hepcidin	
		Ischemia/reperfusion	↑HIF1A	↑HO-1^67^	
			↑HIF1A	↑CD73^72–74^	
	Chronic	Bile duct ligation	↑HIF1A	↑PDGF, ↑FGF^77^	Liver fibrosis
Kidney	Acute	Gentamicin-induced kidney injury	↑HIF1A	↓Caspase-3 activity^83^	
		Ischemic preconditioning	↑HIF1A	↑Bcl-2^85^	
		Ischemia/reperfusion	↑HIF2A	↓VCAM1^87^	
	Chronic	Unilateral ureteral obstruction (UUO)	↑HIF1A	↑Lysyl oxidase^89^	Epithelial–mesenchymal transdifferentiation
		LMB2-induced injury	↑HIF1A	↑COL1A2^90^	Glomerulosclerosis

There are various ways to target the HIF pathway for organ protection (Table 2). HIF activators are mostly used for this purpose, which stabilize HIFs under normal oxygen concentrations^96^. Currently, the most frequently tested method in clinical trials is IPC/IPostC. Remote IPC (RIPC) is conducted by repeated cessation and restoration of blood flow in a particular limb, which is a simple and applicable method to patients undergoing surgery. In a prospective, randomized multicenter trial to observe the effect of RIPC in reducing myocardial IRI, Meybohm et al.^97^ reported that there was no relevant benefits observed in patients. Despite the inconsistency between experimental results from basic science and observations in clinical medicine, RIPC is continuously being studied in humans because of its excellent safety profile and potential^20,85,98^. Pharmacologic activation of HIF by inhibition of PHDs is also an area of interest. Numerous papers have demonstrated the beneficial role of DMOG, which is a competitive inhibitor against 2-oxoglutarate oxygenases including PHDs. Currently, nonspecific PHD inhibitors such as vadadustat, roxadustat, and daprodustat are being tested for treating anemia in CKD patients. Other approaches under investigation are the direct supplementation of HIF downstream target molecules such as EPO or receptor agonists that have been demonstrated to have a role in HIF signaling, such as adenosine receptor agonists. EPO is under trial for its effect in protecting the heart and kidneys from acute injuries, or treating anemia in CKD. Specific adenosine receptor agonists such as neladenoson (ADORA1 agonist) or regadenoson (ADORA2A agonist) are undergoing clinical trials to better understand the effect they may have in organ protection. Interestingly, sevoflurane (fluoromethyl hexafluoroisopropyl ether), an inhalation anesthetic, which is commonly used in clinical practice, has been reported to have preconditioning effects mediated by an increase in HIF1A and a decrease in caspase-3^99^. Preconditioning by sevoflurane is currently undergoing trials for its effect on cardiac IRI protection.

**Table 2:** Current clinical trials related to hypoxia signaling and organ protection

Disease	Study condition	Intervention	Outcome measures	https://ClinicalTrials.gov Identifier (status)
Heart	Postoperative myocardial ischemia in pancreatic surgery	Remote ischemic preconditioning	Postoperative myocardial injury and inflammatory response	NCT03460938 (recruiting)
	Myocardial injury after percutaneous coronary intervention (PCI)	Remote ischemic preconditioning	Troponin	NCT02581618 (completed)
	Myocardial injury after non-cardiac surgery	Remote ischemic preconditioning	Troponin	NCT02427867 (recruiting)
	Perioperative myocardial ischemia in elective operation of abdominal aortic aneurysm	Remote ischemic preconditioning	CKMB and Troponin T	NCT01523262 (unknown)
	Myocardial injury after PCI	Remote ischemic preconditioning	Myocardial injury and cardiac events	NCT00970827 (completed)
	Myocardial damage in patients undergoing coronary artery bypass graft (CABG) surgery	Epoetin-α	Erk1/2, STAT5, Akt and caspase-3, CKMB, Troponin T, NT-proBNP	NCT00524901 (completed)
	Minimally invasive mitral valve (MIMV) repair or replacement	Volatile conditioning (sevoflurane) used during induction (preconditioning)		NCT02551328 (recruiting)
	Adult patient undergoing open heart surgery	Sevoflurane versus ischemic and Sevoflurane preconditioning	Postoperative inotropic score	NCT02715869 (completed)
	Chronic heart failure, NYHA class II–IV	Neladenoson bialanate (BAY1067197, ADORA1 agonist)	LVEF (%), NT-proBNP, Troponin T	NCT02992288 (completed)
Lung	Lung injury during elective lung lobectomy	Remote ischemic preconditioning	Oxidative lung injury	NCT02734654 (completed)
	Lung injury during CABG surgery	Remote Ischemic preconditioning and postconditioning	Pulmonary parameters and plasma IL-6, IL-8, IL-10, TNF-α levels	NCT01144585 (completed)
	Lung transplantation	Regadenoson (ADORA2A agonist)	Activation of iNKT cells, pulmonary graft dysfunction, inflammatory cytokines	NCT03072589 (recruiting)
Liver	Non-alcoholic steatohepatitis (NASH)	CF102 (ADORA3 agonist)	ALT, AST, HDL cholesterol	NCT02927314 (recruiting)
Kidney	Contrast-induced acute kidney injury	Remote ischemic preconditioning	Creatinine, vascular and renal biomarkers	NCT03236441 (recruiting)
	Stage III–IV chronic kidney disease undergoing planned coronary angiography	Remote ischemic preconditioning	Proinflammatory gene expression and anti-inflammatory gene expression in leukocytes, local release of adenosine and NO	NCT02167152 (completed)
	Acute kidney injury in patients undergoing valvular heart surgery	Human recombinant erythropoietin	Incidence of acute kidney injury	NCT01758861 (completed)
	Non-dialysis dependent chronic kidney disease	Vadadustat (PHD inhibitor)	Hemoglobin (Hb)	NCT02648347 (recruiting)
	Chronic kidney disease patients initiating dialysis	Daprodustat (PHD inhibitor)	Hemoglobin (Hb)	NCT03029208 (recruiting)
	Non-dialysis dependent chronic kidney disease	Roxadustat (PHD inhibitor)	Hemoglobin (Hb)	NCT02021318 (active, not recruiting)

As a master regulator of multiple downstream targets, HIF exerts diverse functions and should be altered with caution. This is more perceptible in chronic conditions, where chronic HIF stabilization may have an adverse role. RIPC or nonspecific PHD inhibitors enhance the HIF signaling pathway in a general manner but do not specifically direct its effect. Although this may benefit acute hypoxic conditions such as acute organ injury or organ transplantations, it may lack specificity in certain chronic diseases and lead to unexpected or adverse consequences. Recently, PT2385, a small molecule antagonist of HIF2A, was reported to be effective in treating renal cell cancer. PT2385 specifically inhibits the dimerization of the HIF2A subunit to its partner HIF1B and decrease the expression of HIF2A-dependent genes^100^. Taken together, HIF stabilizers and certain identified downstream target proteins are actively under investigation for its use as treatment options in various clinical settings and their safety profiles are being evaluated. Newer agents targeting the HIF signaling pathway with increased specificity will grant a wider and safer use of these molecules in clinical medicine.

## Concluding remarks

Since the discovery of HIF1A, a hypoxia-inducible transcription factor that binds to the promotor region of *EPO* gene by Gregg Semenza, great progress has been made over the past two decades. In-depth studies on its isotypes and their function, its binding factors/co-activators, and its regulators have been performed. Investigations on HIF downstream signaling cascades have revealed that HIF holds an undisputed biological importance. The advancements in our knowledge are now being actively applied to the bedside—with the hope of yielding novel therapeutics. In the present review, we discussed hypoxia signaling in different diseases and organ systems. Stabilization of HIF by preconditioning/postconditioning or pharmacologic intervention displays a universally protective phenotype across all organs during acute conditions, which is supported by numerous in vivo studies and recent human clinical trials. On the other hand, modulating the HIF pathway in chronic disease conditions is considered more complex, because the effect of HIF stabilization is debatable across different studies. Nonetheless, targeting the HIF signaling pathway in chronic disease conditions still holds promise in effectively managing or delaying the progression of disease. As our understanding of the pathophysiology of diseases and its relation to hypoxia signaling deepens, we will be able to discover additional targets and niches to intervene.
